# HEALTH SURVEILLANCE OF PERSONS LIVING WITH DEMENTIA AND CAREGIVER DYADS

**DOI:** 10.1093/geroni/igac059.1870

**Published:** 2022-12-20

**Authors:** Annie Robitaille, Neil Drummond, Linda Garcia, Himasara Marasinghe, David Barber

**Affiliations:** University of Ottawa, Ottawa, Ontario, Canada; University of Alberta, Edmonton, Alberta, Canada; University of Ottawa, Ottawa, Ontario, Canada; University of Calgary, Calgary, Alberta, Canada; Queen's University, Kingston, Ontario, Canada

## Abstract

The relationship between individuals living with dementia and their caregivers is important and can impact their dementia journey. However, limited national longitudinal data exists about the caregiver-person living with dementia dyad. Availability of such data would provide important information about joint trajectories and help to better identify the needs of caregivers and persons living with dementia across the dementia journey. The objective of this study was to develop a linked national longitudinal database of persons living with dementia and their caregivers. The Canadian Primary Care Sentinel Surveillance Network (CPCSSN) extracts and de-identifies clinical data from electronic medical records (EMR) from approximately 2 million patients across Canada. CPCSSN data is used to identify persons living with dementia and caregivers willing to participate in the study. CPCSSN data from participating dyads are linked (e.g., chronic and mental health conditions, diagnoses, laboratory test results) and additional information about the experiences of persons living with dementia and their caregivers (e.g., ethnicity, amount and type of care provided, burden, availability of support) are collected yearly using surveys. The growing database contains linked, de-identified, comprehensive information about persons living with dementia and their caregivers that will become a rich source of data for researchers, clinicians, and policymakers. Specifics around how the database was developed, and lessons learned will be discussed as these findings can be used as a template to develop similar linked health surveillance databases.

